# Correction: Identification of acrylamide-based covalent inhibitors of SARS-CoV-2 (SCoV-2) Nsp15 using high-throughput screening and machine learning

**DOI:** 10.1039/d5ra90098k

**Published:** 2025-08-22

**Authors:** Teena Bajaj, Babak Mosavati, Lydia H. Zhang, Mohammad S. Parsa, Huanchen Wang, Evan M. Kerek, Xueying Liang, Seyed Amir Tabatabaei Dakhili, Eddie Wehri, Silin Guo, Rushil N. Desai, Lauren M. Orr, Mohammad R. K. Mofrad, Julia Schaletzky, John R. Ussher, Xufang Deng, Robin Stanley, Basil P. Hubbard, Daniel K. Nomura, Niren Murthy

**Affiliations:** a Graduate Program of Comparative Biochemistry, University of California, Berkeley Berkeley CA USA bajajtiya@berkeley.edu; b Innovative Genomics Institute, University of California, Berkeley Berkeley CA USA bmosavati@berkeley.edu; c Department of Bioengineering, University of California, Berkeley Berkeley CA USA nmurthy@berkeley.edu; d Graduate Program of Molecular Toxicology, University of California, Berkeley Berkeley CA USA; e Department of Applied Science and Technology, University of California Berkeley CA USA; f Signal Transduction Laboratory, National Institute of Environmental Health Sciences, National Institutes of Health, Department of Health and Human Services North Carolina USA; g Department of Pharmacology, Li Ka Shing Institute of Virology, University of Alberta Edmonton Alberta Canada; h Department of Physiological Sciences, College of Veterinary Medicine, Oklahoma Center for Respiratory and Infectious Disease, Oklahoma State University Oklahoma USA; i Faculty of Pharmacy and Pharmaceutical Sciences, University of Alberta Edmonton Alberta Canada; j The Henry Wheeler Center for Emerging and Neglected Diseases, University of California, Berkeley Berkeley CA USA; k Department of Chemistry, University of California, Berkeley Berkeley CA USA; l Department of Mechanical Engineering, University of California Berkeley CA USA; m The Molecular Therapeutics Initiative, University of California, Berkeley 344 Li Ka Shing Berkeley CA USA

## Abstract

Correction for ‘Identification of acrylamide-based covalent inhibitors of SARS-CoV-2 (SCoV-2) Nsp15 using high-throughput screening and machine learning’ by Teena Bajaj *et al.*, *RSC Adv.*, 2025, **15**, 10243–10256, https://doi.org/10.1039/D4RA06955B.

The authors regret the following errors in their published article.

Firstly, the sentence “Mohammad S. Parsa was supported by the Natural Sciences and Engineering Research Council of Canada (NSERC).” was omitted from the Acknowledgements section. The corrected Acknowledgements section is as shown below.

**Acknowledgements**

We would like to acknowledge Basil P. Hubbard, Xufang Deng for providing critical suggestions for the manuscript. We would also acknowledge Robert Maxwell from QB3 Mass spectrometry facility for mass spectrometry experiments. The BSL-3 live virus work was supported by the Oklahoma Center for the Advancement of Science and Technology (OCAST) grant HR23096 to Xufang Deng. Niren Murthy would like to acknowledge NIH grants UG3NS115599, R33 and R61DA048444-01, RO1EB029320-01A1, RO1MH125979-01, and funding from the BAKAR Spark award, the Cystic Fibrosis Foundation, and the Innovative Genomics Institute. Julia Schaletzky, and Eddie Wehri were supported by the Henry Wheeler Center for Emerging and Neglected Diseases and through Fastgrants. Robin Stanley would like to acknowledge the in part support by the Intramural Research Program of the NIH, National Institute of Environmental Health Sciences (1ZIAES103340). Mohammad S. Parsa was supported by the Natural Sciences and Engineering Research Council of Canada (NSERC).

Secondly, the affiliations for Dr Niren Murthy should be ^*b*^ and ^*c*^, rather than ^*h*^ as listed in the original article. The corrected author list and contact details are as presented herein.

Finally, a link to https://github.com/msparsa/nsp15-inhibitors-prediction was omitted from the ‘Optimization of AI models’ section of the original main article and the Data availability statement. There was also additional information missing from the Data availability statement, which has been included in the corrected version below.

**Optimization of AI models**

The code for the models that we trained in this paper is available online at https://github.com/bmosavati/AI-Powered-Platform-Drug-Discovery and https://github.com/msparsa/nsp15-inhibitors-prediction.

**Data availability**

The data and code used in this study are available online at https://github.com/msparsa/nsp15-inhibitors-prediction and https://github.com/babakmosavati/AI-Powered-Platform-Drug-Discovery. Mohammad S. Parsa developed the code and notebooks for the final AI/ML pipeline in the manuscript and they are available at the first URL.

The Royal Society of Chemistry apologises for these errors and any consequent inconvenience to authors and readers.

